# Revolutionising multi-sectoral nutrition policy: Insights from the Ethiopian National Information Platform for Nutrition (NiPN) approach

**DOI:** 10.7189/jogh.14.03041

**Published:** 2024-10-18

**Authors:** Taddese Alemu Zerfu, Amare Abera Tareke, Tirsit Genye, Melaku Bayable, Anbissa Muleta, Zekarias Getu, Tarekegn Negese, Hiwot Darsene, Bedassa Tessema, Dejen Tesfaw Molla, Yoseph Halala, Frezer Zewdu, Sisay Sinamo, Daniel Tsegaye, Ingo Neu, Manzura Mirsaidova, Archana Sarkar, Masresha Tessema, Aregash Samuel Hafebo

**Affiliations:** 1 International Food Policy Research Institute (IFPRI), Addis Ababa, Ethiopia; 2 College of Medicine and Health Sciences, Wollo University, Dessie, Ethiopia; 3 Ethiopian Public Health Institute, Addis Ababa, Ethiopia; 4 Jigjiga University, Department of Food Science and Nutrition, Jigjiga, Ethiopia; 5 Federal Ministry of Health, Nutrition Coordination Office, Addis Ababa, Ethiopia; 6 Addis Ababa University, Department of Statistics, Addis Ababa, Ethiopia; 7 Deutsche Gesellschaft für Internationale Zusammenarbeit (GIZ), Bonn, Germany; 8 Deutsche Gesellschaft für Internationale Zusammenarbeit (GIZ), Addis Ababa, Ethiopia

The global nutrition and food security crisis, characterised by troubling trends in various forms of malnutrition ranging from hunger to obesity, has significantly worsened [1,2]. In 2021 and 2022, nearly one-third of the global population faced moderate to severe food insecurity, underscoring a persistent challenge in accessing healthy and sustainable diets [1,3]. This decline in diet quality has led to a surge in malnutrition, with obesity and diet-related noncommunicable diseases (NCDs) reaching epidemic proportions [4–6].

Despite the implementation of numerous nutrition policies and interventions, many low- and middle-income countries (LMICs) continue to grapple with food and nutrition security issues, posing a significant threat to vulnerable populations [3,7]. Food insecurity is particularly evident among the poor, with women and children in rural areas being the most affected [1,2,8]. In 2022 alone, an alarming 230 million children under the age of five experienced some form of malnutrition. Specifically, 148 million (22.3%) were stunted, 45 million (6.8%) were wasted, and 37 million (5.6%) were overweight [1,9,10]. Stunting and wasting were more prevalent in rural areas, while overweight was somewhat more common in urban settings. In Ethiopia, the latest national survey showed that 37% of children under five were stunted, 11% were wasted, and 22% were underweight [11].

Evidence-based approaches and effective programming are essential in tackling these persistent nutrition challenges and improving outcomes. These methods address policy gaps and are cost-effective in resource-poor settings. Recognising the importance of evidence-based policy, driven by political and accountability demands, helps raise awareness and guide decision-making through multisectoral collaboration. However, more evidence does not always mean better policies, as cognitive and institutional factors can hinder effective use. Emphasising knowledge translation in nutrition research is crucial [12], yet many studies focus only on policy formulation, neglecting the systematic analysis of implementation. This highlights the need to address the impact of research on policy and programme execution.

## APPROACHES TO CASE STUDIES

In this study, we employed a case study methodology to evaluate the effectiveness of the National Information Platform for Nutrition (NiPN) in improving evidence-based policymaking in nutrition. By focussing on detailed case studies, we provide in-depth insights into NiPN’s implementation and impact across diverse contexts.

**Figure:**
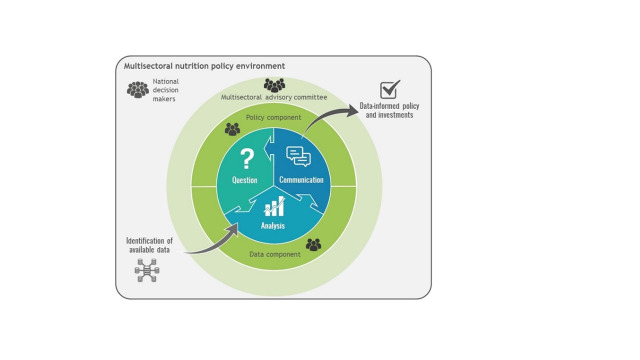
**Photo:** National Information Platform for Nutrition’s approach. Source: National Information Platform for Nutrition. Used with permission.

### Case selection

We selected two cases that collectively illustrate the evidence we are presenting regarding the active implementation of NiPN and its observable policy outcomes. This selection process ensured that the cases offered meaningful insights into NiPN’s effectiveness. Each case was chosen based on the availability of comprehensive data on NiPN’s implementation and outcomes, which was essential for a thorough analysis of the initiative’s impact and scalability.

### Data collection

Data collection was multi-faceted. For one of the cases on school feeding, we conducted a comprehensive review of documents, including policy papers, program reports, and evaluation studies related to NiPN. This review encompassed official communications, funding records, and implementation plans, providing a detailed understanding of NiPN’s operations and impacts.

Additionally, we made observations during various meetings, workshops, and events where NiPN policies and evidence were discussed. This enabled us to document stakeholder interactions and their engagement with NiPN processes.

For the second case study, we employed the Lives Saved Tool (LiST) modeling analysis to estimate various outcomes related to stunting. This included assessing the number of stunting cases averted, additional lives saved, and the reduction in stunting rates. In our analysis, we focussed on three different periods: the initial year of the expansion phase (2021–22), the continued expansion to 700 woredas (2023–25), and the full scale-up of Seqota Declaration (SD) interventions to all 1050 woredas (2026–30). We assessed scenarios with a 10% increase in coverage during these periods. The analysis included data from seven regions and two city administrations, Addis Ababa and Dire Dawa, during the initial expansion phase and expanded to all 11 regions and the two city administrations for subsequent scenarios.

### Data analysis

Qualitative analysis involved content analysis of policy documents and programme reports to evaluate how NiPN addressed evidence gaps and influenced policy changes. Detailed case study reports were prepared, including background information, implementation details, outcomes, and lessons learned. These reports highlighted successes and challenges and offered recommendations for enhancing NiPN’s effectiveness.

Based on these case studies and LiST findings, we developed actionable recommendations to improve evidence-based policymaking and scale NiPN in different contexts. These recommendations focussed on optimising implementation, addressing challenges, and adapting NiPN strategies to regional and contextual needs, aiming to enhance the effectiveness and adaptability of evidence-driven policy solutions.

## RESULTS

### Basics of NiPN

The European Union (EU) introduced NiPN as a strategic initiative designed to improve the alignment between nutrition research and policymaking. This unique evidence-based policy and decision-making approach distinguishes itself by using a reverse methodology; it begins by identifying specific policy needs and then generates targeted evidence to address the needs [13]. It functions through identifying policy gaps, formulating policy questions, conducting targeted research, and disseminating the findings, thereby fostering collaboration to enhance evidence-based policymaking.

Over six years, NiPN has been implemented in nine countries to enhance nutrition data use for evidence-based policy and coordination [13]. It emphasises evidence-based programmes and integration into existing institutions and national multi-sectoral coordination systems for nutrition, using shared data to guide policy, programme design, and resource allocation through a three-phase cycle managed by national structures and partners [14].

### NIPN in Ethiopia

Introduced in 2018, NiPN is hosted by the Ethiopian Public Health Institute (EPHI) and is integrated into the National Nutrition Policy [14]. It receives technical assistance from the International Food Policy Research Institute and the *Deutsche Gesellschaft für Internationale Zusammenarbeit* under the Ethiopian NiPN Technical Assistance Project [15]. In this collaborative framework, NiPN serves as a dynamic platform that engages a wide range of stakeholders, including government agencies, research institutions, non-governmental organisations, and international partners.

### NiPN’s major successes and impact

Over time, NiPN has gained significant interest from various sectors for its role in addressing food and nutrition policy questions. For example, in 2020, Ethiopia’s Federal Ministry of Health asked NiPN to analyse National Nutrition Policy indicators and support the new Food and Nutrition Strategy, highlighting NiPN's importance in policy shaping. Additionally, the Food, Beverage, and Pharmaceutical Development Institute asked NiPN to provide evidence for mandatory food fortification in Ethiopia. Here we explore NiPN’s transformative impact using two illustrative case studies, demonstrating its effectiveness in enhancing evidence-based decision-making in Ethiopia’s food systems and nutrition.

### Case study 1: NiPN’s rapid response to the SD programme

#### Background and context

The SD programme, initiated by the Government of Ethiopia in 2015, aims to eliminate hunger, ensure food security, and enhance nutrition through a multi-phase approach [16]. The programme focusses on reducing child stunting and improving child survival.

#### Implementation

In late April 2023, the EPHI directed a rapid response policy query to NiPN, requesting to evaluate the impact of the SD programme, particularly concerning child stunting and survival. A research team from EPHI and the International Food Policy Research Institute utilised the LiST [17] modelling to assess the effectiveness of the programme.

#### Findings

**Figure 1:**
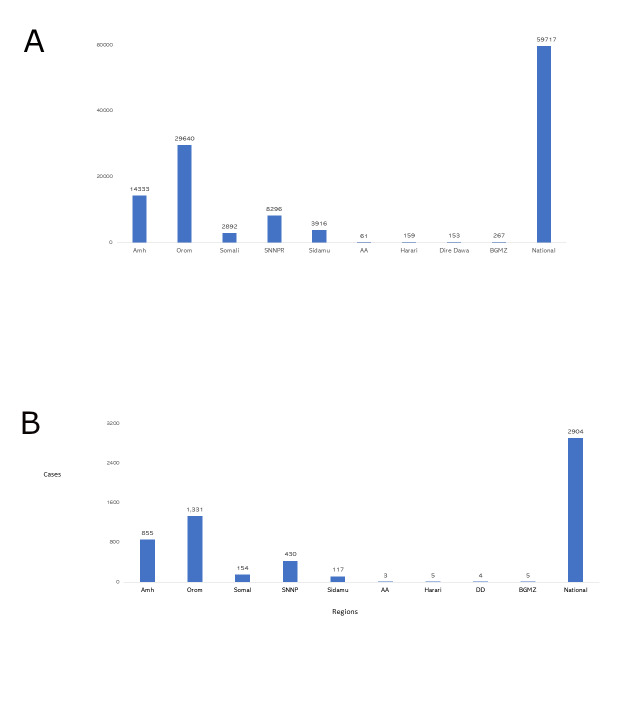
Impact of SD interventions in Ethiopia’s seven regions and two city administrations, 2021–22. **Panel A.** Impact on stunting reduction. **Panel B.** Impact on child mortality.

The analysis found that the SD programme achieved significant outcomes within the initial year of its expansion from 40 to 240 *woredas*, with approximately 60 000 stunting cases being prevented and 2900 child lives saved. These findings were pivotal in justifying further investments and programme adjustments (**Figure 1****,** Panels A and B).

**Figure 2:**
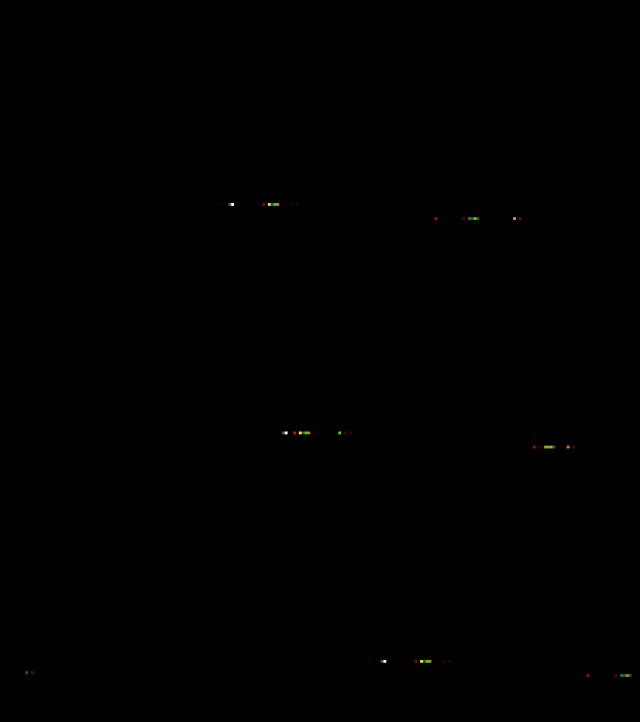
Projected impact of scaling up SD interventions in Ethiopian regions and city administrations, 2023–25. **Panel A.** Impact on stunting. **Panel B.** Impact on child mortality.

Moving forward, forecasts from the LiST model project that extending SD interventions to 700 *woredas* over the next 2–3 years, with a conservative 10% increase in coverage, could prevent approximately 698 892 stunting cases and save 8861 children’s lives (**Figure 2**, Panels A and B). Furthermore, expanding SD interventions to all 1050 *woredas* in Ethiopia could potentially avert up to 5 694 765 stunting cases nationwide by 2030 (**Figure 2**, Panels A and B).

#### Impact and recommendations

The results garnered high praise from key policymakers, including the Deputy Prime Minister and regional leaders. The Government of Ethiopia allocated approximately USD 12.6 million to the SD programme based on these findings. The projected impact was discussed at various national and international forums, including the African Union Summit. The case underscores NiPN’s effectiveness in rapidly responding to policy needs and enhancing nutrition outcomes through evidence-based interventions.

### Case study 2: Empowering nutrition initiatives: NiPN's impact on Ethiopia's school feeding programme

#### Background and context

Ethiopia's school feeding programme (SFP) aims to improve child nutrition and academic performance by providing regular, nutritious meals to students [17]. However, it has faced challenges such as inadequate funding, logistical issues, and varying regional needs [17]. NiPN, with its evidence-based approach, was introduced to address these issues by offering targeted support and strategic guidance.

#### Findings

To address the challenges facing Ethiopia's SFP, NiPN conducted a series of studies, after which we organised a high-level consultative workshop. This event brought together development partners, researchers, and government officials from various ministries, including the Ministry of Health and the Ministry of Education, along with school feeding coordinators. The workshop highlighted achievements and persistent issues, such as lack of administrative structure for SFP, insufficient funding, and technical support. Proposed solutions included diversifying approaches, involving communities through initiatives like school gardens, and forming a national task force to enhance coordination. The workshop marked a shift towards collaborative evidence-sharing, aligning with the SFP team’s needs and recommending new projects for greater sustainability. Discussions also focussed on low-cost, healthy menu options to address regional needs and logistical challenges, aiming to improve the overall effectiveness of the programme.

#### Impact

NiPN's engagement led to substantial enhancements in Ethiopia's school feeding programme. Increased stakeholder collaboration and refined programme strategies resulted in better meal quality and distribution. The initiative also highlighted the importance of integrating local knowledge and adapting strategies to regional contexts. The overall impact includes improved nutrition outcomes for children and greater programme sustainability.

#### Recommendations

To enhance Ethiopia's SFP, it is crucial to secure additional funding to improve the quality of meals and broaden the programme's reach. Strengthening technical support through targeted training and efficient operational guidelines will ensure better programme execution. Engaging local communities by incorporating initiatives such as school gardens can leverage local resources and bolster support for the programme. Additionally, strategies should be adapted to address regional dietary needs and logistical challenges to ensure the programme's effectiveness in various contexts.

#### Overview of NiPN’s evidence-to-policy approach

NiPN’s novel evidence-to-policy approach was showcased through two case studies, highlighting its significant successes. NiPN distinguishes itself by employing a reverse methodology, whereby it first identifies specific policy needs and then generates targeted evidence to address them. This methodology has led to substantial improvements in evidence utilisation and stakeholder engagement, effectively aligning with knowledge utilisation theory that emphasises the systematic application of research findings to decision-making processes, ensuring that evidence is accessible, relevant, and directly applicable to policy development.

#### Comparison with traditional methods

NiPN’s demand-driven model offers a clear contrast to traditional evidence-generation methods. Conventional approaches often involve researchers identifying evidence gaps based on their perspectives, which may not always align with policymakers’ immediate needs [18]. In contrast, NiPN starts by focussing on specific policy requirements, ensuring that the generated evidence is directly relevant and actionable for current policy questions. This model fosters a more effective and responsive approach to policymaking by addressing immediate needs and engaging a diverse range of stakeholders from government agencies, research institutions, non-governmental organisations, and international partners. Such collaboration enhances the practical application of evidence and supports more informed and effective policymaking [19].

#### Case study insights

The case studies demonstrated NiPN's impactful results. In one case, NiPN’s approach led to substantial funding for the SD programme, aimed at eliminating child stunting in Ethiopia by 2030. This achievement underscores NiPN’s ability to mobilise resources and secure high-level political support by aligning evidence with strategic policy goals. In another case, NiPN’s methods resulted in increased stakeholder engagement with national school feeding programmes, illustrating how the approach can effectively enhance the impact of multi-sectoral initiatives. These successes highlight NiPN’s capacity to drive significant improvements in policy outcomes through targeted evidence and strategic stakeholder involvement.

#### Challenges and limitations

Despite its successes, NiPN faces several challenges. The resource-intensive nature of NiPN’s operational cycle and the need for robust stakeholder networks can impede scalability. NiPN’s approach, which involves continuously identifying policy needs, analysing evidence gaps, and formulating policy questions, demands substantial time and expertise, which can be difficult to sustain, especially in resource-constrained settings. Additionally, the effectiveness of NiPN is closely tied to the quality and availability of evidence. In regions with limited data and research infrastructure, generating reliable evidence can be challenging. This dependency on high-quality evidence poses a significant obstacle in areas with weaker research capacities.

#### Adaptation to local contexts

Ethiopia’s unique socioeconomic, cultural, and political conditions significantly influence the outcomes of NiPN’s approach. The high levels of poverty and rural-urban disparities in the country impact the effectiveness of nutrition programmes. NiPN’s success in this context is partly due to its tailored approach, which leverages local knowledge to overcome resource constraints. Culturally, Ethiopia’s diverse ethnic groups and traditional practices shape dietary habits, requiring NiPN’s strategies to be adaptable; politically, its strong government commitment to nutrition and health initiatives, such as the SD, has provided a supportive environment for NiPN’s implementation.

However, these factors also present challenges. The socioeconomic and political conditions that contribute to NiPN’s success in Ethiopia may not be present in other LMICs. Countries with less political stability or lower government support might face difficulties in securing funding and implementing evidence-based policies. Additionally, cultural diversity in Ethiopia might not be as pronounced in other LMICs, which could affect how well NiPN’s approach can be adapted.

#### Future directions and recommendations

To build on NiPN’s success, its model could be adapted to various sectors and regions, offering a way towards strengthening research and data infrastructure and fostering multi-sectoral collaboration. Continuous stakeholder engagement is crucial to maintaining the relevance and adaptability of evidence, ensuring that policy solutions are effective and sustainable. While NiPN’s innovative approach has proven effective in addressing complex challenges such as food insecurity and malnutrition, overcoming challenges related to scalability, resource demands, and evidence quality will be essential for broader implementation and sustained impact.

In conclusion, NiPN’s reverse evidence-to-policy model offers substantial potential for transforming policymaking by prioritising policymakers' needs and ensuring the relevance of evidence. Its approach demonstrates promising applications beyond the nutrition sector, with the possibility for adaptation and expansion to address developmental challenges more effectively.
